# Maternal Circulating Concentrations of Tumor Necrosis Factor-Alpha, Leptin, and Adiponectin in Gestational Diabetes Mellitus: A Systematic Review and Meta-Analysis

**DOI:** 10.1155/2014/926932

**Published:** 2014-08-19

**Authors:** Jie Xu, Yan Hong Zhao, Yun Ping Chen, Xiao Lei Yuan, Jiao Wang, Hui Zhu, Chun Mei Lu

**Affiliations:** ^1^Harbin Medical University, 157 Baojian Road, Nangang District, Harbin, Heilongjiang 150081, China; ^2^First Affiliated Hospital of Harbin Medical University, 199 Dazhi Street, Nangang District, Harbin, Heilongjiang 150001, China; ^3^Second Affiliated Hospital of Harbin Medical University, 148 Baojian Road, Nangang District, Harbin, Heilongjiang 150081, China

## Abstract

Gestational diabetes mellitus (GDM) is one of the most common pregnancy complications. Inflammation may play a role in the pathogenesis of GDM. We performed a systematic review and meta-analysis to determine whether maternal serum concentration of tumor necrosis factor-alpha (TNF-*α*), leptin, and adiponectin were associated with GDM. A systematic search of PubMed and Medline was undertaken. In total, 27 trials were evaluated by meta-analyses using the software Review Manager 5.0. The results showed that maternal TNF-*α* (*P* = 0.0003) and leptin (*P* < 0.00001) concentrations were significantly higher in GDM patients versus controls. However, maternal adiponectin (*P* < 0.00001) concentration was significantly lower in GDM patients compared with controls. Subgroup analysis taking in consideration the effect of obesity on maternal adipokine levels showed that circulating levels of TNF-*α* and leptin remained elevated in GDM patients compared to their body mass index (BMI) matched controls, and adiponectin level remained depressed in GDM individuals. Our findings strengthen the clinical evidence that GDM is accompanied by exaggerated inflammatory responses.

## 1. Introduction

Gestational diabetes mellitus (GDM), which is defined as impaired glucose tolerance with onset or first recognition during pregnancy, is one of the most common pregnancy complications and affects approximately 3–8% of all pregnancies [1, 2]. GDM develops when the maternal insulin supply is not sufficient to compensate for decreased insulin sensitivity during pregnancy. Although the detailed mechanism of how GDM happens remains poorly known, GDM could lead to various adverse outcomes on pregnant women and their offspring, such as gestational hypertension, cesarean delivery, preterm birth, and macrosomia, as well as the predisposition to the development of metabolic syndrome and type 2 diabetes [2].

In recent years, clinical and epidemiological studies have described a clear connection between the development of low-grade inflammatory responses and metabolic diseases, including obesity, type 2 diabetes, and GDM, characterized by secretion of upregulated inflammatory mediators [3]. Particularly inflammatory cytokines have been suspected to be important contributors to the pathogenesis of metabolic dysregulation. Most cytokines, such as tumor necrosis factor-alpha (TNF-*α*), interleukin-6 (IL-6), and leptin, are proinflammatory. Among these proinflammatory cytokines, TNF-*α* and leptin have been suggested as the strongest predictors of pregnancy-associated insulin resistance [4, 5]. One prominent exception is adiponectin, an anti-inflammatory adipokine that promotes insulin sensitization [6].

GDM is thought to be partly attributed to secretion of upregulated inflammatory cytokines from gestational tissues that accelerate insulin resistance [7]. Among these cytokines, extensive attention has been given to TNF-*α*, leptin, and adiponectin. TNF-*α* is one of candidate molecules responsible for causing insulin resistance during pregnancy. It has been suggested that TNF-*α* is a predictor of insulin resistance in human pregnancy [4]. During late pregnancy, TNF-*α* is inversely correlated with insulin sensitivity. Neutralization of the TNF-*α* signaling leads to an improvement in insulin sensitivity [8]. Leptin plays a key role in the energy intake and energy expenditure and is said to have proinflammatory activities. In healthy pregnancies, the maternal serum leptin level is in a negative linear correlation with the head circumference of the newborns. In GDM pregnancies, an inverse relationship is shown between the body length, head circumference, and body weight of the newborns and the maternal leptin concentration [9]. Increased leptin levels may contribute to insulin resistance in GDM and in the third trimester of normal pregnancy [9]. On the other hand, adiponectin, one of a smaller number of anti-inflammatory factors, is thought to have beneficial effects on insulin sensitivity and anti-inflammation. It stimulates glucose uptake in skeletal muscle by activating AMP-activated protein kinase [10], and administration of adiponectin to diabetic mice has been shown to enhance insulin activity [11]. In previously published studies, circulating levels of leptin, adiponectin, and TNF-*α* in the early pregnancy closely predict the development of GDM.

Many studies have reported on the maternal serum concentrations of cytokines in GDM patients. However, conflicting results are available in the literature about the association of cytokines and GDM. To our knowledge, there remains a lack of systematic reviews and meta-analysis on the relationship between GDM and maternal circulating concentrations of cytokines. Therapeutic strategies based on imbalance of proinflammatory and anti-inflammatory cytokines for preventing or treating metabolic dysfunction in GDM should be based on evidence.

In the present study, we did a systematic review and meta-analysis of published data in accordance with the preferred reporting items for systematic reviews and meta-analysis (PRISMA) statement [12]. Our objective was to address the association of maternal circulating levels of cytokines (TNF-*α*, leptin, and adiponectin) and GDM.

## 2. Methods

### 2.1. Literature Search and Data Extraction

Our research protocol included the detailed research question, search strategy, and screening criteria. The detailed research question was composed of the patient, intervention, comparator, outcome, and study design (PICOS) approach.

The search data sources were Pubmed and Medline between 1966 and 2012.1 The search key words consisted of “gestational diabetes mellitus or GDM,” “tumor necrosis factor alpha,” “TNF-*α*,” “leptin,” and “adiponectin.” In addition, all references cited in the found studies were reviewed to identify additional studies.

After screening the database search results, full-text assessment was done for study selection. The following information was extracted into a computer-based spreadsheet: authors, year of publication, and clinical data. For continuous outcomes, information on the numbers of cases and controls, maternal plasma mean concentrations of TNF-*α*, leptin, and adiponectin, and standard difference (SD) was also abstracted. Jie Xu and Yan Hong Zhao established the research protocol, and database searches were conducted independently by Yun Ping Chen and Xiao Lei Yuan. Any inconsistencies were resolved by discussions with the 3rd reviewer, Jiao Wang.

### 2.2. Inclusion Criteria

The PICOS research question was a foundation for study selection. Eligible studies had to meet the following criteria: (1) with cytokine concentrations as the exposure and GDM as the outcome, either cross-sectional or prospective case controlled design is accepted for concentration detection studies; (2) the study should contain original data; (3) maternal blood was the object or one of the objects of study for concentration detection, and maternal blood was collected in the late-second or third trimester of pregnancy; (4) for concentration detection, plasma concentrations of TNF-*α*, leptin, or adiponectin for GDM and control groups were available or could be provided as mean (±SD). GDM was diagnosed if patients met at least two of the following four diagnostic criteria [37]: fasting plasma glucose (FPG) ≥ 95 mg/dL; glucose level at 1, 2, and 3 hours after meals ≥180 mg/dL, ≥155 mg/dL, and ≥140 mg/dL, respectively. Controls were normal glucose tolerance pregnant women. Thus, review articles, records published in languages other than English, and studies measuring cytokine concentrations from placenta, peripheral blood cells, amnionic fluid, cord blood, or serum sample following stimulation or sample collected in the nonpregnant period were excluded due to the lack of comparability. Moreover, studies with case-only or with data unclear or provided in forms other than mean (±SD) were ruled out as methodological reason.

### 2.3. Quality Assessment

The quality of the primary studies was assessed using Newcastle-Ottawa Quality Assessment Scale (NOS) with some modifications to match the needs of this study. Items assessed included three items: patient selection, comparability of GDM and control groups, and assessment of exposure. A study can be awarded a maximum of one star for each numbered item within the selection and exposure categories. A maximum of two stars can be given for comparability. Studies were graded on an ordinal star scoring scale with higher scores representing studies of higher quality. The quality of each study was graded as either level 1 (0 to 5) or level 2 (6 to 9) [38].

### 2.4. Publication Bias

Publication bias was assessed by a Funnel plot asymmetry test.

### 2.5. Evaluation of Statistic Association

We performed power calculations with G∗Power program version 3.0 [39]. Statistics were performed with Review Manager 5.0 (Cochrane Collaboration, Oxford, United Kingdom). The inverse variance method was adopted for continuous data meta-analysis with a weighted mean difference (WMD) and 95% CI. The level of heterogeneity between studies was tested graphically on Forest plots and statistically using Cochran's chi-square analysis and indicated intuitively by an *I* ^2^ index. *I* ^2^> 50% suggested heterogeneity. As recommended by Song et al. [40], *P* < 0.1 was used as the cut-off for significance of heterogeneity and using random effects model; otherwise, fixed effects model was used.

## 3. Results

### 3.1. Quality Control

**Figure 1:**
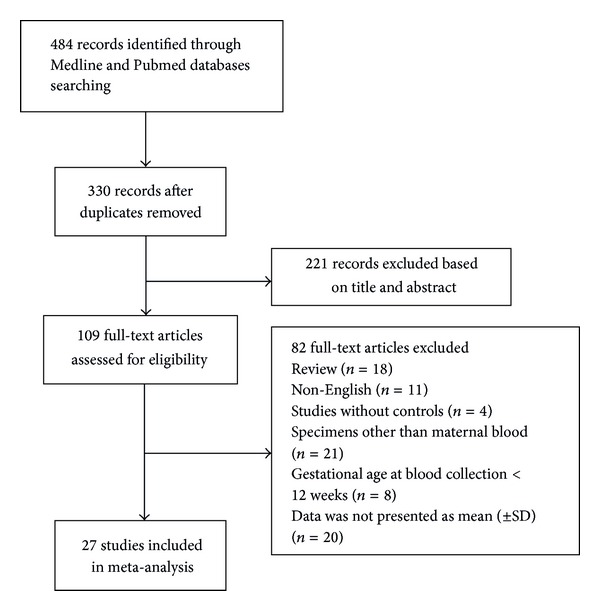
Flow diagram of the selection and systematic review of studies.

Database search identified 484 potentially relevant records, of which 109 full-text articles were assessed for eligibility (Figure 1). In total, 82 articles were excluded for the reasons given in Figure 1. Thus, only 27 studies were used for meta-analysis, which included 10 for TNF-*α* concentrations [4, 7, 9, 13–19], 18 for leptin concentrations [4, 7, 9, 14, 15, 17, 18, 20–30], and 15 for adiponectin concentrations [13, 14, 16–18, 22, 23, 25, 29, 31–36]. The characteristics of the trials included in the meta-analysis were summarized in Table 1.

The quality assessment and scores of these studies were presented in Table 2. Among these studies, 11 studies had a quality score of 7 [4, 9, 14, 20, 21, 28, 30–32, 34, 35], 14 studies scored 6 [7, 13, 15–19, 22–24, 26, 27, 29, 36], and 2 studies scored 5 [25, 33], which illustrated that the methodological quality was generally good.

The power of our sample size for meta-analysis of continuous outcome to detect correlation between TNF-*α*, leptin, and adiponectin maternal serum level and GDM was 95.0%, 99.6%, and 99.7% (*α* = 0.05, effect size index = 0.2, small effect convention for continuous data), respectively.

### 3.2. Heterogeneity and Publication Bias

For TNF-*α*, leptin, and adiponectin concentration detection, different studies used enzyme-linked immunosorbent assay (ELISA) kits from different suppliers. Significant heterogeneity was observed in all sub-meta-analyses (Table 3). This justified the adoption of random effects model in all analyses. Publication bias was not observed except in the outcome of TNF-*α* concentration meta-analysis, as demonstrated by the funnel plots (see Supplementary Material available online at http://dx.doi.org/10.1155/2014/926932).

### 3.3. Serum TNF-*α* Concentration and GDM

**Figure 2:**
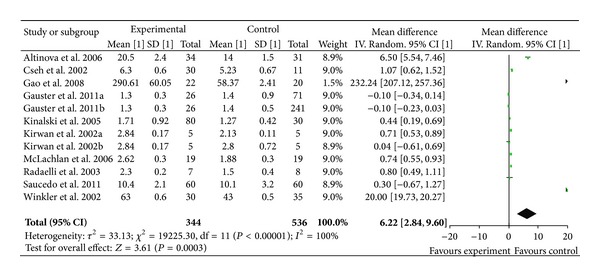
Mean difference (MD) and 95% CI of individual studies and pooled data for the association of maternal concentration of TNF-*α* with GDM risk. Positive values denote higher in GDM patients; negative values denote higher in healthy control subjects.

Meta-analysis of maternal TNF-*α* level was comprised of 10 studies with 12 comparisons, as Gauster et al. and Kirwan et al. divided the control subjects into lean and obese group. There was significantly elevated TNF-*α* concentration in serum of GDM patients versus normal pregnancies with an overall WMD of 6.22 pg/mL (95% CI [2.84, 9.60], *P* = 0.0003) (Figure 2).

Because obesity is associated with insulin resistance and an increased risk of GDM, we also examined the effect of obesity on plasma TNF-*α* level. We categorized the studies according to their design into two classes with body mass index (BMI) matched and BMI not matched between control and GDM. Plasma TNF-*α* concentrations remained significantly higher in GDM patients compared to their BMI matched control subjects (*P* = 0.002, [WMD] = 2.08 pg/mL, 95% CI [0.75, 3.41] pg/mL).

### 3.4. Serum Leptin Concentration and GDM

**Figure 3:**
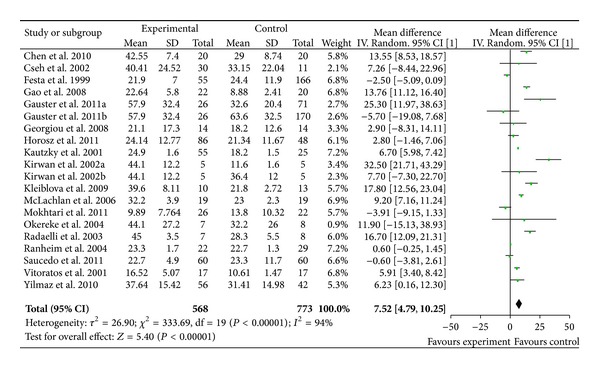
Mean difference (MD) and 95% CI of individual studies and pooled data for the association of maternal concentration of leptin with GDM risk. Positive values denote higher in GDM patients; negative values denote higher in healthy control subjects.

Leptin measurements of 568 GDM patients and 773 controls were extracted from 18 studies with 20 comparisons, as Gauster et al. and Kirwan et al. divided the control subjects into lean and obese group. GDM patients had significantly higher serum leptin concentration with an overall WMD of 7.52 ng/mL (95% CI [4.79, 10.25], *P* < 0.00001) (Figure 3). We also assessed the effect of BMI on maternal leptin level by subgroup analysis. Plasma leptin concentration remained significantly elevated in GDM patients compared to their BMI matched control subjects (*P* < 0.00001, [WMD] = 7.14 ng/mL, 95% CI [4.00, 10.28] ng/mL).

### 3.5. Serum Adiponectin Concentration and GDM

**Figure 4:**
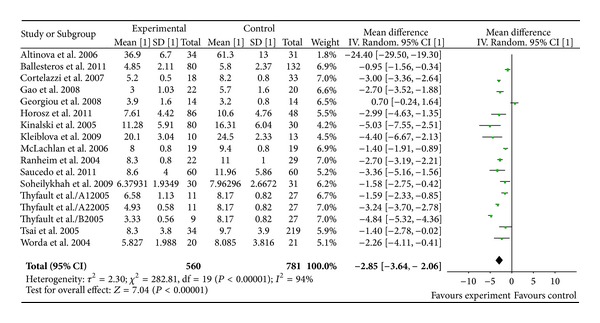
Mean difference (MD) and 95% CI of individual studies and pooled data for the association of maternal concentration of adiponectin with GDM risk. Positive values denote higher in GDM patients; negative values denote higher in healthy control subjects.

Adiponectin measurements of 560 GDM patients and 781 controls were extracted from 15 studies with 17 comparisons, as Thyfault et al. divided the GDM patients into three groups. There was a significantly decreased adiponectin level in GDM patients compared to controls with an overall WMD of −2.85 *μ*g/mL (95% CI [−3.64, −2.06], *P* < 0.00001) (Figure 4). Subgroup analysis showed that serum adiponectin concentration remained significantly lower in GDM patients compared to their BMI matched control subjects (*P* < 0.00001, [WMD] = −2.66 *μ*g/mL, 95% CI [−2.85, −2.48]  *μ*g/mL).

## 4. Discussion

To the best of our knowledge, this is the first systematic review to address the correlation of maternal serum concentrations of three cytokines (TNF-*α*/leptin/adiponectin) and GDM. Findings of the meta-analysis confirmed increased levels of TNF-*α* and leptin and a decreased level of adiponectin in GDM patients compared with normal pregnancies, suggesting that imbalance in the expression of pro- and anti-inflammatory cytokines may contribute to impaired glucose homeostasis in GDM.

Inflammatory cytokines, including TNF-*α*, IL-6, and IL-8, have been involved in the pathogenesis of insulin resistance. Among these inflammatory cytokines, the evidence that insulin resistance is linked to TNF-*α*, but not IL-6 and IL-8, is well established. Although type 2 DM is associated with IL-6 polymorphism [41] and higher plasma concentrations of IL-6 [42], there is no direct evidence for an association between IL-6 expression and pregnancy-induced insulin resistance. Additionally, TNF-*α* has been demonstrated to be the most significant predictor of pregnancy-induced insulin resistance and be more highly synthesized and released from the placenta compared with IL-6 or IL-8 [43]. Hence, TNF-*α* is more likely to exert crucial effects on insulin resistance during pregnancy.

Adipokines, such as leptin, adiponectin, and resistin, may also be involved in the pathogenesis of insulin resistance. Although the leptin is produced mainly by adipocytes, there is strong evidence that the placenta, rather than maternal adipose tissue, contributes to the rise in maternal leptin concentrations during pregnancy [44]. Pregnancy is considered a leptin resistant state; circulating leptin levels are two- to threefold higher concentrations as compared to nonpregnancy condition. Results on circulating leptin in patients with GDM have been inconsistent. Thus, levels of the leptin are not altered in patients with GDM as compared to healthy pregnant women in some reports [18, 23], whereas other authors demonstrate elevated levels of leptin in women with GDM [9, 14]. And a strong linear correlation between increased maternal plasma leptin and increased risk of GDM has been found [14]. Resistin, an adipocyte-derived cytokine, is poorly produced by the placenta [45]. Despite elevated resistin levels in GDM, the independent relationship between insulin resistance and circulating resistin concentrations cannot be established [46]. Adiponectin, one of a smaller number of anti-inflammatory factors, is considered to have beneficial effects on insulin sensitivity. Low adiponectin serum levels are demonstrated to be linked with type 2 DM and insulin resistance [47]. Moreover, Lain et al. show that women with low levels of first trimester adiponectin are more likely to be diagnosed with GDM as compared to women with higher adiponectin levels [48], suggesting that downregulation of adiponectin in the first trimester of pregnancy might be a predictor of GDM. In view of these findings, leptin and adiponectin are likely to be more important in the pathogenesis of pregnancy-associated insulin resistance as compared to resistin.

TNF-*α*, leptin, and adiponectin have been demonstrated to be produced in placenta [49, 50]. In vitro, most of the placental TNF-*α* and leptin are released into the maternal circulation, which contributes to the rise in maternal TNF-*α* and leptin concentrations during pregnancy; little is released to the fetal side [4]. Release of TNF-*α* and leptin from placenta during pregnancy is considered to be a diabetogenic factor exacerbating insulin resistance. TNF-*α* is reported to increase with gestational progression and to be strongly associated with insulin sensitivity in normal pregnancy [4]. When placentas obtained from GDM patients are cultured under high glucose conditions, the accumulation of TNF-*α* in media is significantly greater compared with placentas incubated in normal glucose concentrations [51]. Animal models characterized by reduced-leptin signaling show hyperphagia, obesity, and insulin resistance [52], and leptin management improves insulin sensitivity and glucose metabolism in these models [53]. Additionally, positive leptin relation to fasting glucose and insulin was shown in studies in the presence of elevated leptin in GDM. Adiponectin is thought to have beneficial effects on insulin sensitivity and anti-inflammatory activities. Investigators have reported that depressed maternal adiponectin concentrations, measured in early pregnancy or at delivery, were found in GDM women compared with nondiabetic pregnant women. Therefore, it is reasonable to speculate that these cytokines can be a cause of impaired glucose metabolism in GDM. That may indicate possible cytokines influence on fetal growth.

GDM is characterized by an amplification of the low-grade inflammation already existing in normal pregnancy [54]. The data presented in this study demonstrate that the maternal proinflammatory cytokines, TNF-*α* and leptin, are elevated in GDM patients as compared to normal pregnancy; but the anti-inflammatory adiponectin is depressed, which strengths the clinical evidence that GDM is accompanied by exaggerated inflammatory response. Increased circulating concentrations of TNF-*α* cause a chronic inflammatory environment and enhance leptin production. Conversely, leptin increases the production of TNF-*α* and IL-6 by monocytes [55] and stimulates the production of CC-chemokine ligands [56]. Thus, a vicious circle develops, resulting in an aggravated inflammatory situation, which might worsen metabolic dysfunction in GDM. Furthermore, TNF-*α* and other proinflammatory mediators suppress the production of adiponectin by adipocytes [57]. Because of the insulin-sensitizing effects, low levels of adiponectin might further aggravate insulin resistance in GDM. To summarise, GDM may arise in part from an amplification of inflammatory situation. It is the upregulation of proinflammatory mediators, that is, TNF-*α* and leptin, and the downregulation of anti-inflammatory molecules, that is, adiponectin, that lead to the development of chronic inflammatory state and contribute to the hyperinsulinemia in GDM.

It is well known that obesity is strongly associated with inflammation, which contributes to insulin resistance [3]. Many of the studies included in the meta-analysis found that patients with GDM had significantly higher TNF-*α* and leptin concentrations and lower adiponectin concentrations than control women. The differences remained statistically significant after adjusting for BMI in some studies, but not all. Moreover, some studies found a significant positive correlation between BMI values and levels of TNF-*α* and leptin and an inverse correlation between BMI and adiponectin levels in GDM [9, 14, 16, 33]. So, subgroup analysis taking in consideration the effect of BMI values on maternal cytokine levels was performed in this meta-analysis. We categorized the studies according to their designs into two classes with respect to BMI matched and BMI not matched between controls and GDM groups. We found that plasma TNF-*α* and leptin concentrations remained significantly elevated in GDM patients compared to their BMI matched control subjects, and adiponectin concentration remained significantly depressed. These data suggest that maternal weight in GDM seems to have less important role in modifying cytokine levels.

Limitations of this meta-analysis should be acknowledged. First, studies on cytokine concentrations and GDM that provided results in form other than mean (±SD) were ruled out in our analysis as methodological reason, although most of them observed higher median cytokine levels in GDM patients compared with control subjects. Second, concentrations provided by the included studies vary greatly with a maximum of 200-fold discrepancy between that detected by Gao et al. and that detected by Gauster et al. High degree of heterogeneity in three outcomes of concentration meta-analysis is not surprising due to different assay kits, procedures, operations for cytokine detection, and other unperceived variables. Although regression analyses to further explore sources of heterogeneity were not conducted in our study because of the power limitation of software, variety in assay kits was considered to have central contribution to the heterogeneity. Therefore, random effects model was adopted in our meta-analysis as it gives a larger *P* value and wider confidence intervals. Third, some factors that can alter maternal serum cytokine levels (e.g., maternal weight, smoking, insulin therapy, and labor onset) and other confounding factors cannot be excluded. Finally, it is very important to keep publication bias in mind when meta-analysis relies on previously published studies because positive results are more likely to be published than negative results.

## 5. Conclusion

This meta-analysis confirmed the increased levels of TNF-*α* and leptin and the decreased level of adiponectin in GDM, suggesting that increase in proinflammatory cytokines and decrease in anti-inflammatory factors may contribute to impaired glucose homeostasis in GDM and indicating that these cytokines might be of predictive value in GDM diagnosis. However, further studies are required to investigate the mechanism of the alteration of the three cytokines.

## Supplementary Material

The plot in supplementary material resembles a symmetrical inverted funnel (the 95%CI), inside which are all the studies included in the meta-analysis. This is a scatter plot of the treatment effects estimated from individual studies on the horizontal axis (mean difference, MD), against a measure of study size on the vertical axis (SE *[*MD*]*).

## Figures and Tables

**Table 1:** Characteristics of included studies.

SNP ID/author	GDM/control	Age (years)	BMI (kg/m^2^)	Insulin therapy/diet control	Insulin (mIU/mL)	Gestational age at blood collection
TNF-*α*						
Altinova et al. [13]	34/31	GDM 31.5 ± 4.3; control 29.0 ± 4.9	GDM 29.5 ± 3.5; control 28.0 ± 4.3	Insulin therapy: 5 Diet control: 29	GDM 58.7 ± 15.5; control 36.9 ± 5.8	Third trimester
Cseh et al. [9]	30/11	GDM 28.00 ± 2.70; control 26.00 ± 2.60	GDM 33.40 ± 6.40; control 25.40 ± 2.60	Insulin therapy: all	NP	Third trimester
Gao et al. [14]	22/20	GDM 30.97 ± 3.64; control 28.80 ± 2.62	GDM 23.92 ± 3.51; control 21.83 ± 2.33	Insulin therapy: yes, but not all	NP	GDM 29.28 ± 2.79 w; control 28.00 ± 3.09 w
Gauster et al. [15]	26/241	GDM 29.2 ± 6.2; obese-control 27.6 ± 5.6; lean-control 28.5 ± 6.0	GDM 35.8 ± 1.5; lean-control 22.2 ± 3.5; obese-control 34.6 ± 7.4	Insulin therapy: yes, but not all	GDM 30.6 ± 20.3; lean-control 12.5 ± 6.4; obese-control 23.3 ± 13.1	GDM 38.5 ± 0.7 w; control 38.7 ± 0.6 w
Kinalski et al. [16]	80/30	GDM 29.0 ± 4.9; control 28.2 ± 6.0	GDM 27.11 ± 4.58; control 25.61 ± 3.41	Insulin therapy: no Diet control: all	GDM 11.03 ± 12.18; control 5.46 ± 2.02	Third trimester
Kirwan et al. [4]	5/10	GDM 29 ± 2; lean-control 33 ± 2; obese-control 30 ± 1	GDM 30.8 ± 2.8; obese-control 27.3 ± 2.4; Lean-control 19.8 ± 1.0	NP	Obese-GDM 27.5 ± 5.6; lean-control 10.7 ± 0.9	Between 34 and 36 w
McLachlan et al. [17]	19/19	GDM 33 ± 1; control 33 ± 1	GDM 31.5 ± 1.3; control 31.6 ± 1.3	Insulin therapy: 5 Diet control: 14	NP	Third trimester
Radaelli et al. [7]	7/8	NP	GDM 39.7 ± 2.6; control 34.0 ± 3.3	Insulin therapy: 7	GDM 58.7 ± 15.5; control 36.9 ± 5.8	GDM 38.5 ± 0.5 w; control 38.9 ± 0.4 w
Saucedo et al. [18]	60/60	GDM 31.9 ± 5.6; control 24.8 ± 6.4	GDM 30.2 ± 4.9; control 28.4 ± 7.3	Insulin therapy: yes, but not all	GDM 61.1 ± 40.3; control 50.7 ± 33.3	At 30 w
Winkler et al. [19]	30/35	GDM 28.0 ± 2.7; control 26.0 ± 2.6	GDM 33.4 ± 6.4; control 25.4 ± 2.6	Insulin therapy: all	NP	GDM 27.6 ± 6.1 w; control 22.5 ± 10.8 w
Leptin						
Chen et al. [20]	20/20	GDM 31.2 ± 6.3; control 28.7 ± 5.3	GDM 29.2 ± 5.8; control 27.8 ± 4.0	NP	GDM 24.12 ± 14.4; control 15.95 ± 7.1	GDM 37.1 ± 2.3 w; control 39.0 ± 1.7 w
Cseh et al. [9]	30/11	GDM 28.00 ± 2.70; control 26.00 ± 2.60	GDM 33.40 ± 6.40; control 25.40 ± 2.60	Insulin therapy: all	NP	Third trimester
Festa et al. [21]	55/166	GDM 29.4 ± 5.9; control 26.4 ± 5.2	GDM 28.8 ± 4.7; control 25.9 ± 4.3	Insulin therapy: no Diet control: all	GDM 67.0 ± 34.7; control 59.2 ± 35.8	GDM 26.0 ± 5.0 w; control 25.4 ± 1.5 w
Gao et al. [14]	22/20	GDM 30.97 ± 3.64; control 28.80 ± 2.62	GDM 23.92 ± 3.51; Control 21.83 ± 2.33	Insulin therapy: yes, but not all	NP	GDM 29.28 ± 2.79 w; control 28.00 ± 3.09 w
Gauster et al. [15]	26/241	GDM 29.2 ± 6.2; obese-control 27.6 ± 5.6; lean-control 28.5 ± 6.0	GDM 35.8 ± 1.5; lean-control 22.2 ± 3.5; obese-control 34.6 ± 7.4	Insulin therapy: yes, but not all	GDM 30.6 ± 20.3; lean-control 12.5 ± 6.4; obese-control 23.3 ± 13.1	GDM 38.5 ± 0.7 w; control 38.7 ± 0.6 w
Georgiou et al. [22]	14/14	GDM 33.8 ± 5.0; control 32.6 ± 3.4	GDM 28.2 ± 8.4; control 24.7 ± 5.1	Insulin therapy: 6 Diet control: 8	GDM 14.3 ± 8.3; control 8.9 ± 3.7	At 28 w
Horosz et al. [23]	86/48	GDM 31.8 ± 4.3; control 30.7 ± 3.5	GDM 28.39 ± 5.32; control 25.40 ± 2.72	Insulin therapy: yes, but not all	GDM 9.77 ± 4.94; control 8.10 ± 3.32	Between 27 and 32 w
Kautzky-Willer et al. [24]	55/25	GDM 30.9 ± 0.9; control 29.6 ± 1.9	GDM 28.0 ± 0.9; control 28.1 ± 0.8	Insulin therapy: yes, but not all	GDM 127.2 ± 13.2; control 72.0 ± 12.3	At 28 w
Kirwan et al. [4]	5/10	GDM 29 ± 2; lean-Control 33 ± 2; obese-Control 30 ± 1	GDM 30.8 ± 2.8; obese-control 7.3 ± 2.4; lean-control 19.8 ± 1.0	NP	Obese-GDM 27.5 ± 5.6; lean-control 10.7 ± 0.9	Between 34 and 36 w
Kleiblova et al. [25]	10/13	GDM 34.6 ± 2.5; control 33.1 ± 1.3	GDM 30.1 ± 2.3; control 22.7 ± 0.9	Insulin therapy: all	GDM 144.8 ± 64.56; control 23.4 ± 1.96	GDM 268.9 ± 4.2 d; control 276.9 ± 1.6 d
McLachlan et al. [17]	19/19	GDM 33 ± 1; control 33 ± 1	GDM 31.5 ± 1.3; control 31.6 ± 1.3	Insulin therapy: 5 Diet control: 14	NP	Third trimester
Mokhtari et al. [26]	26/22	GDM 32.69 ± 6.85; control 28.18 ± 8.93	GDM 28.51 ± 3.66; control 27.24 ± 4.06	NP	NP	Third trimester
Vitoratos et al. [27]	17/17	GDM 33 ± 2.3; control 34 ± 2.7	GDM 36.74 ± 6.52; control 34.86 ± 5.91	NP	GDM 21.29 ± 10.16; control NP	At 29 w and 33 w
Okereke et al. [28]	7/8	GDM 29.9 ± 4.1; control 31.6 ± 3.4	GDM 28.7 ± 6.3; control 26.2 ± 4.5	Insulin therapy: all	GDM 0.046 ± 0.02; control 0.089 ± 0.03	Between 34 and 36 w
Radaelli et al. [7]	7/8	NP	GDM 39.7 ± 2.6; control 34.0 ± 3.3	Insulin therapy: all	GDM 58.7 ± 15.5; control 36.9 ± 5.8	GDM 38.5 ± 0.5 w; control 38.9 ± 0.4 w
Ranheim et al. [29]	22/29	NP	GDM 31.4 ± 1; control 28.3 ± 0.6	Insulin therapy: 30% Diet control: 70%	GDM 389 ± 84; control 75 ± 6	GDM 38.1 ± 0.3 w; control 38.5 ± 0.3 w
Saucedo et al. [18]	60/60	GDM 31.9 ± 5.6; control 24.8 ± 6.4	GDM 30.2 ± 4.9; control 28.4 ± 7.3	Insulin therapy: yes, but not all	GDM 61.1 ± 40.3; control 50.7 ± 33.3	At 30 w
Yilmaz et al. [30]	56/42	GDM 31.45 ± 4.92; control 28.1 ± 4.02	GDM 29.01 ± 4.93; control 28.02 ± 4.27	NP	GDM 14.62 ± 12.04; control 8.81 ± 4.28	GDM 30.85 ± 3.39 w; control 31.2 ± 3.69 w
Adiponectin						
Altinova et al. [13]	34/31	GDM 31.5 ± 4.3; control 29.0 ± 4.9	GDM 29.5 ± 3.5; control 28.0 ± 4.3	Insulin therapy: 5 Diet control: 29	GDM 58.7 ± 15.5; control 36.9 ± 5.8	Third trimester
Ballesteros et al. [31]	80/132	GDM 31.88 ± 5.19; control 31.33 ± 4.86	GDM 25.56 ± 4.94; control 24.83 ± 5.13	Insulin therapy: 29 Diet control: 51	GDM 10.03 (7.01–15.12); control 7.73 (5.83–13.28)	GDM 27.51 ± 1.41 w; control 27.46 ± 1.40 w
Cortelazzi et al. [32]	18/33	GDM 34 ± 4.5; control 29 ± 5.2	GDM 24.7 ± 2.1; control 21.8 ± 1.2	Insulin therapy: 10 Diet control: 8	NP	Between 37 and 41 w
Gao et al. [14]	22/20	GDM 30.97 ± 3.64; control 28.80 ± 2.62	GDM 23.92 ± 3.51; control 21.83 ± 2.33	Insulin therapy: yes, but not all	NP	GDM 29.28 ± 2.79 w; control 28.00 ± 3.09 w
Georgiou et al. [22]	14/14	GDM 33.8 ± 5.0; control 32.6 ± 3.4	GDM 28.2 ± 8.4; control 24.7 ± 5.1	Insulin therapy: 6 Diet control: 8	GDM 14.3 ± 8.3; control 8.9 ± 3.7	At 28 w
Horosz et al. [23]	86/48	GDM 31.8 ± 4.3; control 30.7 ± 3.5	GDM 28.39 ± 5.32; control 25.40 ± 2.72	Insulin therapy: yes, but not all	GDM 9.77 ± 4.94; control 8.10 ± 3.32	Between 27 and 32 w
Kinalski et al. [16]	80/30	GDM 29.0 ± 4.9; control 28.2 ± 6.0	GDM 27.11 ± 4.58; control 25.61 ± 3.41	Insulin therapy: no Diet control: yes	GDM 11.03 ± 12.18; control 5.46 ± 2.02	Third trimester
Kleiblova et al. [25]	10/13	GDM 34.6 ± 2.5; control 33.1 ± 1.3	GDM 30.1 ± 2.3; control 22.7 ± 0.9	Insulin therapy: all	GDM 144.8 ± 64.56; control 23.4 ± 1.96	GDM 268.9 ± 4.2 d; control 276.9 ± 1.6 d
McLachlan et al. [17]	19/19	GDM 33 ± 1; control 33 ± 1	GDM 31.5 ± 1.3; control 31.6 ± 1.3	Insulin therapy: 5 Diet control: 14	NP	Third trimester
Ranheim et al. [29]	22/29	NP	GDM 31.4 ± 1; control 28.3 ± 0.6	Insulin therapy: 30% Diet control: 70%	GDM 389 ± 84; control 75 ± 6	GDM 38.1 ± 0.3 w; control 38.5 ± 0.3 w
Saucedo et al. [18]	60/60	GDM 31.9 ± 5.6; control 24.8 ± 6.4	GDM 30.2 ± 4.9; control 28.4 ± 7.3	Insulin therapy: yes, but not all	GDM 61.1 ± 40.3; control 50.7 ± 33.3	At 30 w
Soheilykhah et al. [33]	30/31	Matched	Matched	NP	GDM 148.52 ± 258.30; control 84.67 ± 35.16	At 28 w
Thyfault et al./A1 [34]	11/27	GDM 29.9 ± 1.8; control 26.1 ± 1.1	GDM 35.1 ± 2.3; control 33.4 ± 1.5	NP	GDM 11.5 ± 2.1; control 15.9 ± 3.3	GDM 39.5 ± 0.2 w; control 39.2 ± 0.3 w
Thyfault et al./A2 [34]	11/27	GDM 30.7 ± 1.7; control 26.1 ± 1.1	GDM 39.9 ± 2.5; control 33.4 ± 1.5	NP	GDM 28.7 ± 6.9; control 15.9 ± 3.3	GDM 39.1 ± 0.4 w; control 39.2 ± 0.3 w
Thyfault et al./B [34]	9/27	GDM 29.1 ± 1.7; control 26.1 ± 1.1	GDM 39.6 ± 3.0; control 33.4 ± 1.5	NP	GDM 65.5 ± 31.8; control 15.9 ± 3.3	GDM 38.0 ± 0.6 w; control 39.2 ± 0.3 w
Tsai et al. [35]	34/219	GDM 32.4 ± 3.9; control 30.9 ± 4.1	GDM 27.0 ± 3.4; control 25.4 ± 2.9	Insulin therapy: no Diet control: all	GDM 121.0 ± 45.3; control 89.2 ± 36.3	GDM 27.0 ± 3.4 w; control 25.4 ± 2.9 w
Worda et al. [36]	20/21	GDM 34.3 ± 4.5; control 29.4 ± 6.2	GDM 25.6 ± 4.6; control 24.4 ± 4.6	Insulin therapy: all	NP	GDM 32.1 ± 2.5 w; control 32.2 ± 4.1 w

BMI, body mass index; TNF-*α*, tumor necrosis factor-alpha; w, weeks; NP, not provided; data are presented as mean + standard error of the mean or *n* (%).

**Table 2:** Assessment of study quality.

Authors	Year	Selection				Comparability	Exposure			Score
		1	2	3	4	5	6	7	8	
Cseh et al. [9]	2002	∗	∗		∗	∗∗	∗	∗		∗∗∗∗∗∗∗
Gao et al. [14]	2008	∗	∗		∗	∗∗	∗	∗		∗∗∗∗∗∗∗
Kirwan et al. [4]	2002	∗	∗	∗	∗	∗∗		∗		∗∗∗∗∗∗∗
Chen et al. [20]	2010	∗	∗		∗	∗∗	∗	∗		∗∗∗∗∗∗∗
Festa et al. [21]	1999	∗	∗		∗	∗∗	∗	∗		∗∗∗∗∗∗∗
Okereke et al. [28]	2004	∗	∗	∗	∗	∗∗	∗	∗		∗∗∗∗∗∗∗
Yilmaz et al. [30]	2010	∗	∗		∗	∗∗	∗	∗		∗∗∗∗∗∗∗
Ballesteros et al. [31]	2011	∗	∗		∗	∗∗	∗	∗		∗∗∗∗∗∗∗
Cortelazzi et al. [32]	2007	∗	∗		∗	∗∗	∗	∗		∗∗∗∗∗∗∗
Thyfault et al. [34]	2005	∗	∗		∗	∗∗	∗	∗		∗∗∗∗∗∗∗
Tsai et al. [35]	2005	∗	∗		∗	∗∗	∗	∗		∗∗∗∗∗∗∗
Altinova et al. [13]	2007	∗			∗	∗∗	∗	∗		∗∗∗∗∗∗
Gauster et al. [15]	2011	∗			∗	∗∗	∗	∗		∗∗∗∗∗∗
Kinalski et al. [16]	2005	∗			∗	∗∗	∗	∗		∗∗∗∗∗∗
McLachlan et al. [17]	2006	∗			∗	∗∗	∗	∗		∗∗∗∗∗∗
Radaelli et al. [7]	2003	∗	∗			∗∗	∗	∗		∗∗∗∗∗∗
Ranheim et al. [29]	2004	∗			∗	∗∗	∗	∗		∗∗∗∗∗∗
Saucedo et al. [18]	2011	∗			∗	∗∗	∗	∗		∗∗∗∗∗∗
Winkler et al. [19]	2002	∗			∗	∗∗	∗	∗		∗∗∗∗∗∗
Georgiou et al. [22]	2008	∗	∗			∗∗	∗	∗		∗∗∗∗∗∗
Horosz et al. [23]	2011	∗			∗	∗∗	∗	∗		∗∗∗∗∗∗
Kautzky-Willer et al. [24]	2001	∗			∗	∗∗	∗	∗		∗∗∗∗∗∗
Mokhtari et al. [26]	2011	∗	∗		∗	∗	∗	∗		∗∗∗∗∗∗
Vitoratos et al. [27]	2001	∗			∗	∗∗	∗	∗		∗∗∗∗∗∗
Worda et al. [36]	2004	∗	∗		∗	∗	∗	∗		∗∗∗∗∗∗
Kleiblova et al. [25]	2010	∗				∗∗	∗	∗		∗∗∗∗∗
Soheilykhah et al. [33]	2009	∗			∗	∗∗		∗		∗∗∗∗∗

**Table 3:** Summary of heterogeneity of these meta-analyses.

Outcome	*N* (GDM, control)	Heterogeneity *χ* ^2^ (*P* value)	Inconsistency *I* ^2^ (%)	Analysis model
TNF-*α* level	880 (344, 536)	19225.30 (*P* < 0.00001)	100	Random model
Leptin level	1341 (568, 773)	333.69 (*P* < 0.00001)	94	Random model
Adiponectin level	1341 (560, 781)	282.81 (*P* < 0.00001)	94	Random model
