# Supplementary material for: Maternal Circulating Concentrations of Tumor Necrosis Factor-Alpha, Leptin, and Adiponectin in Gestational Diabetes Mellitus: A Systematic Review and Meta-Analysis
Source: ScientificWorldJournal. 2014 Aug 19;2014:926932. doi: 10.1155/2014/926932 (PMC4151523; doi:10.1155/2014/926932)
Supplement: Supplementary file 1 — The plot in supplementary material resembles a symmetrical inverted funnel (the 95%CI), inside which are all the studies included in the meta-analysis. This is a scatter plot of the treatment effects estimated from individual studies on the horizontal axis (mean difference, MD), against a measure of study size on the vertical axis (SE [MD]). [file 926932.f1.ppt]

## Slide 1
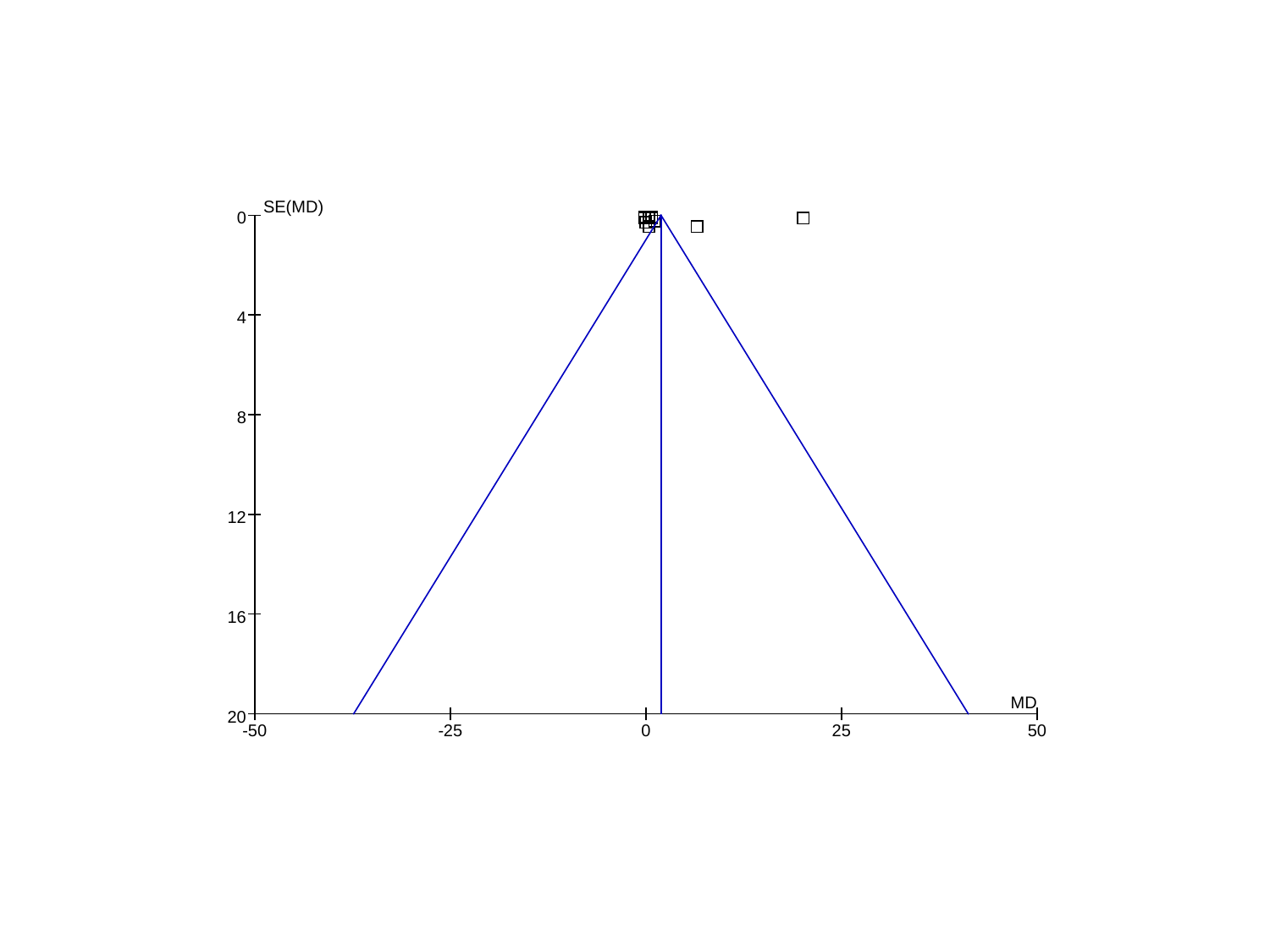

TNF-α and GDM
Fig 5. Funnel plot showing the association between Standard Error of mean difference [SE(MD)] and mean difference (MD) as it was calculated by the fixed effect model.

## Slide 2
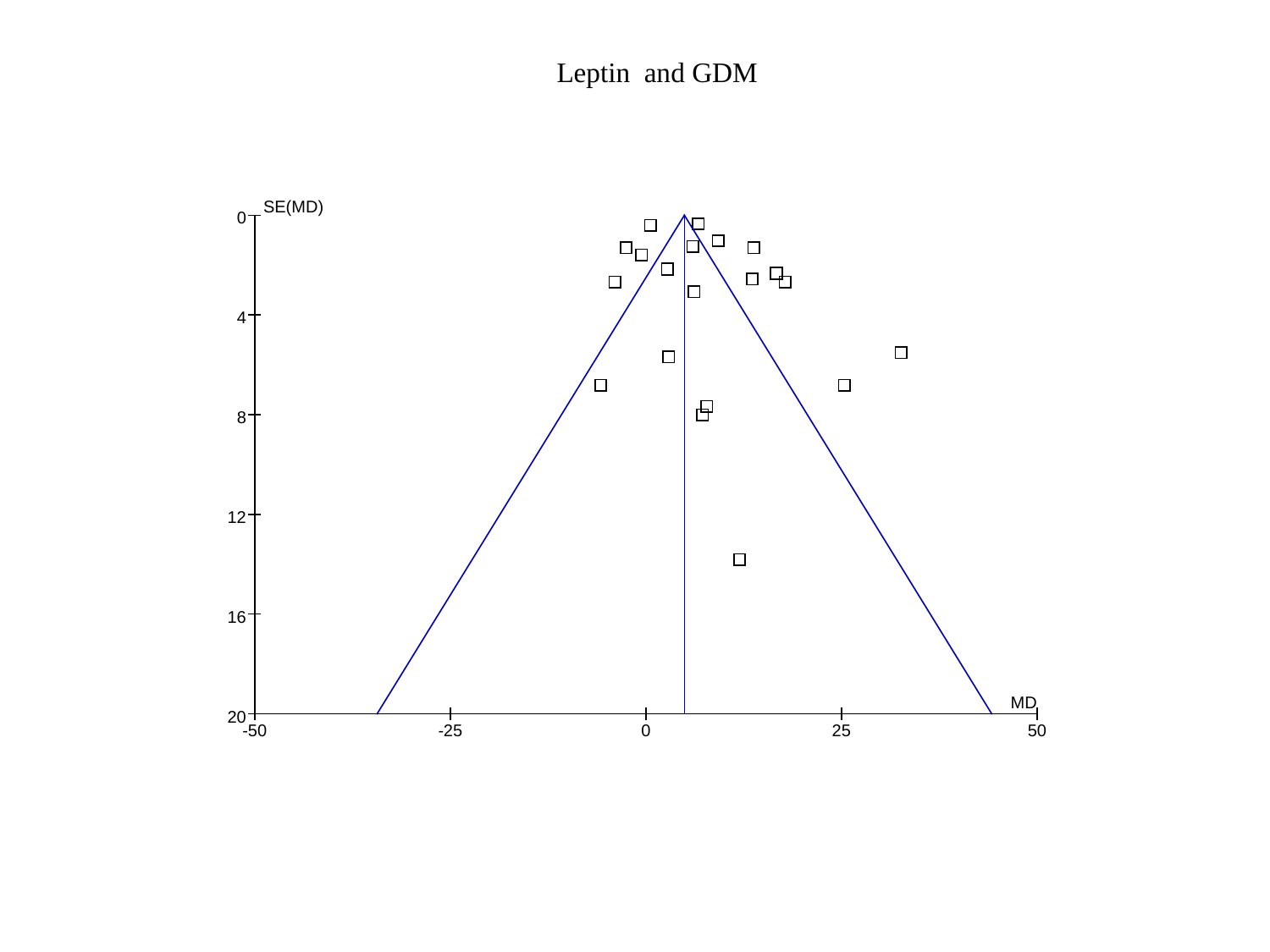

Leptin and GDM
Fig 6. Funnel plot showing the association between Standard Error of mean difference [SE(MD)] and mean difference (MD) as it was calculated by the fixed effect model.

## Slide 3
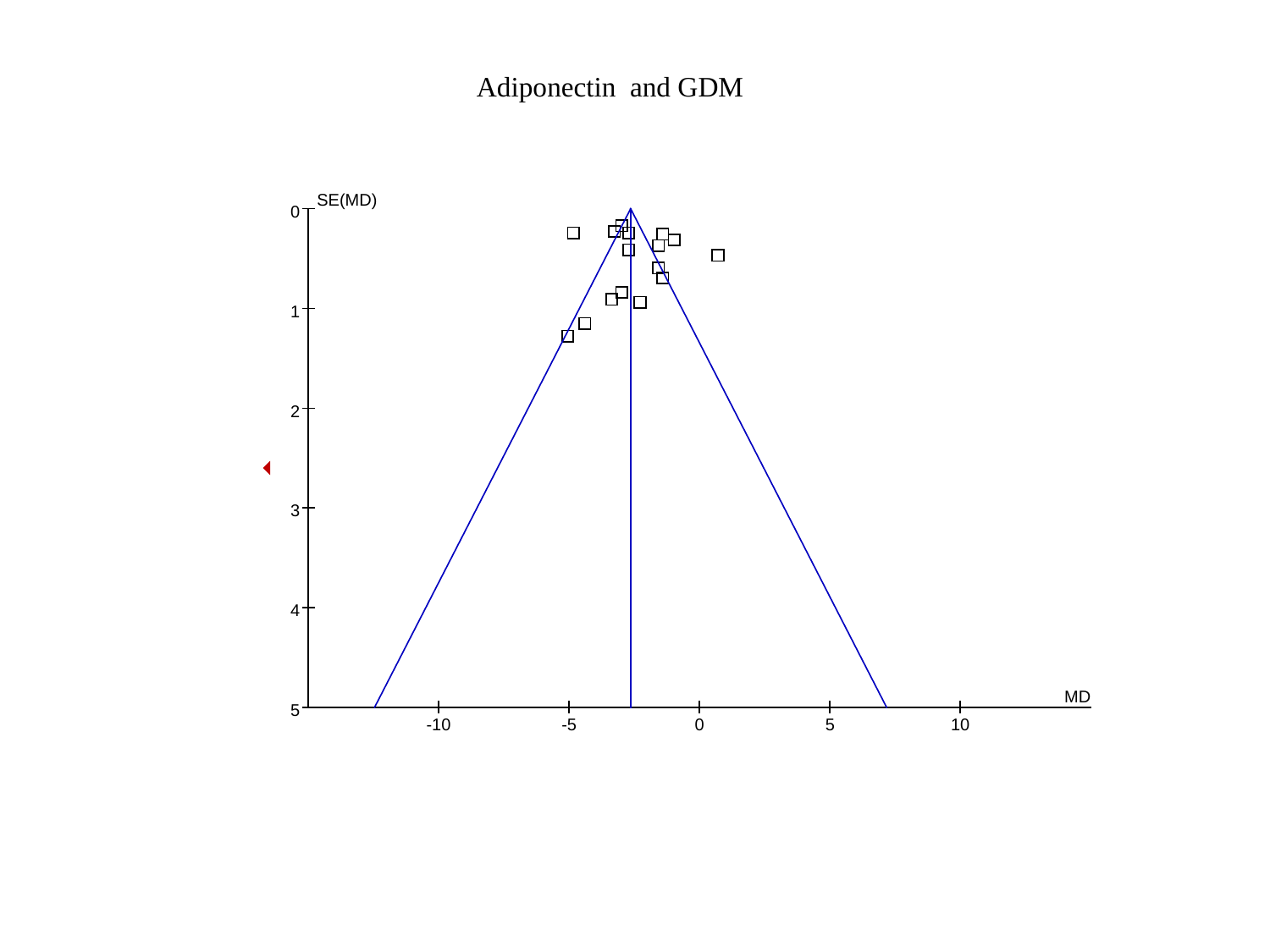

Adiponectin and GDM
Fig 7. Funnel plot showing the association between Standard Error of mean difference [SE(MD)] and mean difference (MD) as it was calculated by the fixed effect model.
